# Biomimetic Three-Dimensional (3D) Scaffolds from Sustainable Biomaterials: Innovative Green Medicine Approach to Bone Regeneration

**DOI:** 10.3390/jfb16070238

**Published:** 2025-06-29

**Authors:** Yashaswini Premjit, Merin Lawrence, Abhishek Goyal, Célia Ferreira, Elena A. Jones, Payal Ganguly

**Affiliations:** 1 School of Food Science and Nutrition, University of Leeds, Leeds LS2 9JT, UK; 2 School of Biological and Chemical Sciences, University of Galway, H91W2TY Galway, Ireland; 3 Deallus Consulting, Gurugram 122002, Haryana, India; 4 Leeds Institute of Rheumatic and Musculoskeletal Medicine, Faculty of Medicine and Health, University of Leeds, Leeds LS7 4SA, UK; 5 Leeds Biomedical Research Centre, Leeds Teaching Hospitals NHS Trust, Leeds LS7 4SA, UK

**Keywords:** biomimetics, 3D scaffolds, natural polymers, mesenchymal stromal cells, tissue engineering, bone repair, biocompatibility, tissue regeneration

## Abstract

Bone repair and regeneration following an injury still present challenges worldwide. Three-dimensional (3D) scaffolds made from various materials are used for bone tissue engineering (BTE) applications. Polymers, minerals and nanotechnology are now being used in combination to achieve specific goals for BTE, including the delivery of antimicrobials through the scaffolds to prevent post-surgical infection. While several materials are utilised for BTE, natural polymers present a unique set of materials that can be manipulated to formulate scaffolds for BTE applications. They have been found to demonstrate higher biocompatibility, biodegradability and lower toxicity. Some even naturally mimic the bone microarchitecture, providing inherent structural support for BTE. Natural polymers may be simply classified as those from plant and animal sources. From both sources, there are different types of proteins, polysaccharides and other specialised materials that are already in use for research in BTE. Interestingly, these have the potential to revolutionise the field of BTE with a sustainable approach. In this review, we first discuss the different natural polymers used in BTE from plant sources, followed by animal sources. We then explore novel materials that are aimed at sustainable approaches, focusing on innovation from the last decade. In these sections, we outline studies of these materials with different types of bone cells, including bone marrow mesenchymal stromal cells (MSCs), which are the progenitors of bone. We finally outline the limitations, conclusions and future directions from our perspective in this dynamic field of polymers in BTE. With this review, we hope to bring together the updated existing knowledge and the potential future of innovation and sustainability in natural polymers for biomimetic BTE applications for fellow scientists, researchers and surgeons in the field.

## 1. Introduction

In the last few decades, the field of bone tissue engineering (BTE) has gained much importance for applications in bone regeneration and repair. It is a unique field that has emerged as a section of tissue engineering (TE), focusing specifically on the regeneration and repair of bone after trauma, tumour resection or after damage due to systemic bone diseases like osteoporosis (OP) [1,2]. The process of bone regeneration and repair after trauma poses significant challenges worldwide. This process may become less effective if patients have complex fractures or in patients with other co-morbidities like diabetes [3,4,5]. Between 2019 and now, around 180 million new cases of bone fractures have been recorded, with OP in women potentially leading to 9 million annual cases worldwide [5,6]. Impaired healing and increased vulnerability towards fractures often lead to non-union, requiring multiple surgical interventions with increased direct and indirect medical costs [7].

Surgeries are invasive, often necessitate follow-up procedures within a couple of years and can lead to post-operative infections. Given these challenges in bone repair, interest in the field of BTE has grown significantly. This promising field, with further optimisation, has the potential to benefit patients with bone defects [8,9]. Thus, there exists a medical gap, highlighting the need to harness BTE’s capabilities to address medical, economic and societal impacts of bone damage and defects.

There are four major elements of BTE rather accepted by all—scaffolds, osteogenic cells, growth factors and mechanical environment. The factor of mechanical environment for the regeneration of weight-bearing bones becomes crucial, also known as the diamond concept [10,11]. More recently, two more factors: vasculature and host factors, have been recognised as additional crucial elements for bone TE. The overall concept is adapted to the type of bone and the extent of damage that needs to be repaired. For osteogenic cells, bone progenitor mesenchymal stromal cells (MSCs) are commonly used due to their regeneration potential, their ability to facilitate bone formation and their involvement in regulating bone remodelling in vivo [12,13,14]. For a successful BTE application, a scaffold with MSCs and growth factors must be placed in the optimal environment in order to enable the formation of the bone tissue on the scaffolds.

This comes with its own set of challenges. Among the biggest challenges is identifying the right material for the scaffold that promotes osteointegration, has minimal immune reaction when implanted in the host, and degrades in a timely fashion [15]. The material must be safe in vitro (not cytotoxic) and in vivo (biocompatible), must be stable for long enough to support the growth of cells into bone tissues, while being biodegradable without forming any harmful metabolites. Another challenge is related to the requirement of the scaffold material to ideally be resistant to infections, which recently has gained a lot of attention. This is a consideration in cases of open fractures with a high risk of infection and osteomyelitis. For this purpose, several types of metallic, non-metallic and drug nanoparticles with anti-bacterial properties have been investigated by scientists in the field [16,17]. Above all, the scaffold, along with all the elements, must be able to mimic the conditions in vivo to provide the right environment for bone regeneration—also referred to as ‘biomimetics’ in BTE.

**Figure 1:**
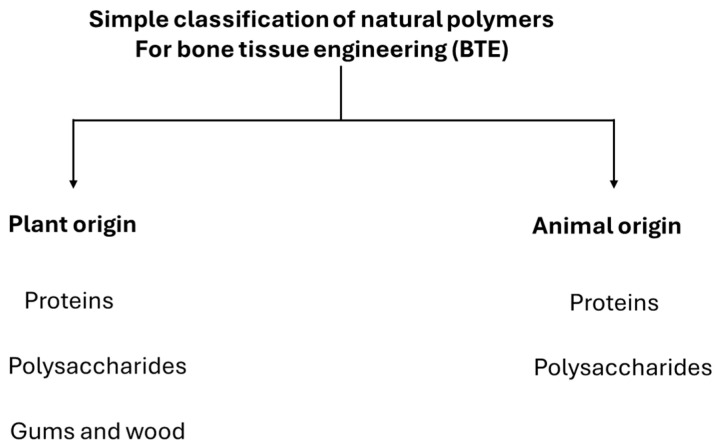
Simple classification of natural polymers for BTE applications discussed in this review article.

The main requirement for bone scaffolds is their mechanical strength. Thus, the first generation of implants was purely metallic (titanium-based). The second generation of implants was ceramics-based, including hydroxyapatite and tri-calcium phosphate or animal decellularised bone. [18,19]. Recently, some of the commonly used materials for scaffolds in BTE have included natural polymers, semi-synthetic and synthetic polymers, metallic scaffolds, bio-glass and ceramics [19]. While polymers on their own typically do not provide with mechanical strength required for BTE, they have several properties that make them an ideal choice of material in combination with others for BTE. Specifically, natural polymers have been found to provide better biocompatibility, have higher biodegradability and have lower levels of toxicity in comparison to the other types of materials used for BTE [20,21,22]. Very simply put, natural polymers can be classified as those sourced from plant and from animal sources (Figure 1). Some of these also provide structural properties that are able to mimic the intricate microstructure within bone and aid bone formation [23,24,25].

**Figure 2:**
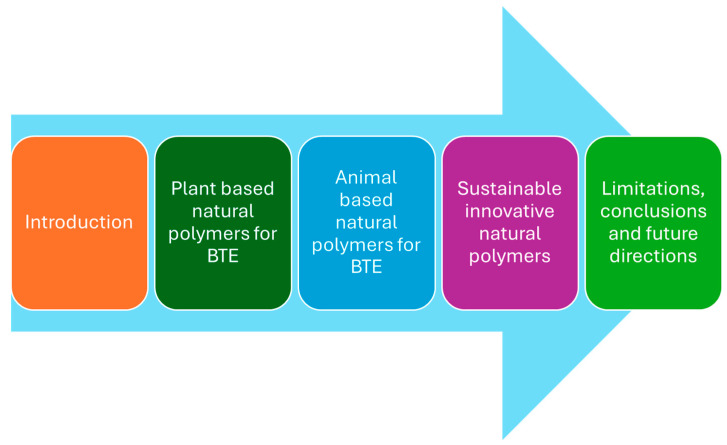
Graphical image of the contents of the review.

In this review, we aim to outline the different types of natural materials that have been used for BTE applications and the progress made in the field in the last five years. This is then segregated into natural polymers from plant and animal sources. Next, we specifically focus on studies that investigated novel naturally occurring materials with a sustainable approach. We finally discuss the limitations, conclusions and future directions of the field (Figure 2). With the recent advances in this field dissected in our review, we hope that the scientific, medical, pharmaceutical and biomedical communities globally will collaborate and innovate to help patients with bone defects.

## 2. Plant-Based Natural Polymers for BTE

Plant-derived biomaterials have gained considerable interest in BTE primarily due to their abundance and alignment with the principles of green and sustainable medicine. As the field advances towards environmentally friendly solutions, the concept of ‘green scaffolds’ for BTE has become increasingly relevant with the use of biomaterials designed with sustainability, biocompatibility, and ethical sourcing [26,27]. Plant-based proteins and polysaccharides serve as ideal candidates for green scaffold designs, offering a biodegradable, low-immunogenic alternative to synthetic and/or animal-derived materials [28,29]. Furthermore, biodegradable polymers are often preferred in BTE for repairing and healing tissues, as they tend to accelerate the treatment process, while simultaneously eliminating the need for implant removal surgery [30].

Plant-based scaffolds are structurally versatile, easy to process and scalable, thereby supporting their translation to clinical and industrial applications. Furthermore, their degradation products are usually non-toxic and non-inflammatory, contributing to a safer in vivo profile [31,32]. The biomimetic potential of plant-derived scaffolds has positioned them as promising tools for regenerative strategies [28]. In the following Sections, we delve into recent advances in the use of plant-based proteins and polysaccharides in BTE, evaluating their potential in next-generation green biomaterial design.

### 2.1. Proteins

In recent years, plant-derived proteins have gained prominence as promising candidates for scaffold fabrication in BTE, mainly for their intrinsic biocompatibility, biodegradability, and ease of processing [33]. Proteins such as zein (from corn) [34], soy protein [35], and wheat gluten [36] have demonstrated structural integrity and biocompatibility suitable for supporting cell adhesion and proliferation. The exploration of plant-based protein scaffolds offers a unique intersection between materials science, sustainability, and regenerative medicine, paving the way for scalable alternatives in bone repair strategies, owing to their compatibility with mammalian cell culture systems [37,38].

#### 2.1.1. Wheat Gluten

Wheat gluten (WG), a proteinaceous biopolymer sourced from *Triticum aestivum*, has gained significant interest in BTE [39]. Comprising approximately 85% of the total protein content, WG is traditionally extracted by washing wheat flour dough to eliminate starch and soluble components. This yields a viscoelastic protein matrix primarily consisting of gliadins and glutenins, which are part of the prolamin family [36,38,40]. Gliadins are monomeric peptides (28–70 kDa), whereas glutenins create polymeric structures interconnected by disulfide linkages, enhancing the network’s elasticity [36,39].

The mechanical and biological properties of WG are fundamental to its effectiveness as a scaffold material. Its hydrophobic structure creates a stable network via H-bonding and disulfide linkages, providing elasticity and structural integrity, which are important for BTE [40,41]. Moreover, its amino acid composition, containing residues equivalent to those present in collagen, enhances its significance in scaffold construction, especially in replicating native bone extracellular matrix (ECM) [40]. Despite its inherent insolubility, chemical alterations such as enzymatic hydrolysis or acid/alkali treatment may improve its solubility and modify its functionality for biomedical applications [38]. WG foams have been reported to degrade in aqueous alkaline conditions within five weeks [42].

Recent research has proven WG’s efficacy in diverse composite scaffold systems. Ramakrishnan et al. reported that the integration of WG into nano-hydroxyapatite (nHA)–gelatin–silica composite scaffolds markedly improved thermal crosslinking, thereby facilitating the adhesion, proliferation and osteogenic differentiation of MSCs over a 21-day duration [40]. In their rat calvarial defect model, scaffolds containing WG with pore sizes ranging between 100 and 300 μm boosted new bone formation, hence confirming their osteoconductive and osteo-integrative potential.

WG has been successfully employed to improve injectable bone cements. Fan et al. demonstrated that β-tricalcium phosphate (β-TCP) cements modified with bioglass (BG), WG and poly (γ-glutamic acid) exhibited significant improvements in mechanical characteristics and biological function [43]. The compressive strength of the formulation, including WG, rose from 8.8 MPa to 53.1 MPa after 5 days. Furthermore, these formulations displayed remarkable cytocompatibility with MC3T3-E1 cells, indicating their suitability for load-bearing bone defect repair.

Zhao et al. further enhanced the applicability of WG by integrating it into a polybutylene succinate (PBS)/magnesium phosphate (MP) scaffold [44]. This ternary PMWC composite had a highly porous structure ranging between 400 and 600 μm with both macro- and micropores, which improved apatite mineralisation and in vitro degradability. In vivo, the addition of WG significantly improved new bone formation and vascularisation, as confirmed by synchrotron radiation microcomputed tomography (SRmCT) and immunohistochemical analysis. These findings reveal that WG seems to act as a key modulator of scaffold bioactivity and osteogenesis.

Further exploring unconventional applications, Holmes et al. examined the use of bread-based scaffolds, composed mostly of WG [41]. The inherently porous (300–500 μm pore size) WG crumb structure appeared to promote mammalian cell proliferation in vitro, indicating a unique and effective pathway for scaffold construction.

These research findings highlight the immense potential of WG in the development of plant-based scaffolds for BTE [40,41,44]. The fibrous protein structure, inherent crosslinking ability and tunable solubility allow the manipulation of its mechanical properties, cellular interactions and degradation behaviour [38,39,40]. Its compatibility with various biomaterials, including bioactive glasses, calcium phosphates [43,44], synthetic polymers, and natural matrices [38,41], highlight its importance in multicomponent scaffold systems aimed at osteoconductive, osteoinductive, as well as angiogenic responses.

#### 2.1.2. Zein Protein

Zein, a storage protein obtained from maize (*Zea mays*), has emerged as a viable plant-derived biomaterial for BTE. Since it is biodegradable and derived from renewable sources, zein offers an attractive alternative to synthetic and animal-derived polymers [29,45]. It has been certified as Generally Recognised as Safe (GRAS) by the U.S. Food and Drug Administration (FDA) and has been extensively used in drug delivery and coating applications [29,46].

Zein is biochemically composed of four protein subclasses: α, β, γ, and δ-zein, with α-zein being the predominant variant [47]. This subclass exhibits several advantages such as flexibility, biocompatibility, low toxicity and biodegradability, rendering zein an excellent candidate for scaffold fabrication and regenerative medicine [26]. Its high content of hydrophobic amino acids such as leucine and proline contributes to favourable antioxidant and mild antimicrobial properties [48]. Furthermore, zein exhibits strong adherence to hydroxyapatite (HA) and facilitates cell adhesion and proliferation, characteristics essential for osteoconductive scaffolds [29,47]. The degradation rate of zein protein for 12 weeks was reported to be 48.10% and 17.00% in vivo and in vitro, respectively [49].

A significant area of research pertains to the development of zein-based fibrous scaffolds. Limaye et al. demonstrated that zein promotes deep cellular infiltration, improved MSC proliferation and promotes angiogenesis, as evidenced by increased CD31 (PECAM-1 or platelet endothelial cell adhesion molecule) expression. The fibrous structure (fibre diameter of gelatin: 1.25  ±  0.50 μm and zein: 1.44  ±  0.55 μm) offers a large surface area-to-volume ratio that is essential for nutrient exchange and tissue ingrowth [29]. These findings confirm zein’s function not merely as a passive scaffold but also as an active support system for vascularised tissue formation.

The potential of zein can be substantially increased in composite systems. Ranjbar et al. created a bioactive glass (58S)-based scaffold covered with kaempferol-infused zein, demonstrating a substantial enhancement in mechanical strength (from 0.88 MPa to 3.06 MPa) [45]. In vitro and in vivo studies demonstrated a pore size of 200–500 μm, elevated ALP activity, enhanced osteogenic gene expression, and bony island formation when co-cultured with BM MSCs. This work demonstrates the dual benefits of mechanical reinforcement and bioactive delivery facilitated by zein.

Similarly, Zaersabet et al. employed a salt-leaching technique to construct 3D zein scaffolds incorporated with nano-hydroxyapatite (nHA). At a concentration of 12.5 wt% nHA, the compressive modulus (reaching 79.1 MPa) and ultimate strength (2.7 MPa) were enhanced, making it suitable for load-bearing applications. The mean pore size was 345 ± 84.5 μm for zein and 307 ± 76.1 μm for zein/nHA scaffolds. The degradation rates on day 5 were 20%, and 11% in zein and zein/nHA scaffolds, respectively. On day 30, degradation of zein and zein/nHA scaffolds reached 48.6% and 40%, respectively. The zein/nHA scaffolds also showed an enhanced expression of essential osteogenic genes (*Runx2*, *ALP*, *Col1A1*), suggesting significant osteogenic differentiation potential in C2C12 cells. The scaffold’s porosity (61–70%) and biodegradability significantly enhanced its suitability for bone regeneration [37].

In a different composite strategy, Plath et al. employed an alternative method by integrating zein into poly(ε-caprolactone) (PCL) to fabricate electrospun nanofibres with improved hydrophilicity, mechanical strength and bactericidal characteristics. Increasing the concentration of zein to 40 wt% significantly lowered the water contact angle (from 118° to 73°), enhanced Young’s modulus (from 260 MPa to 980 MPa) and facilitated MSC adhesion and spreading [50]. The average fibre diameter ranged from 200 to 400 nm. The zein coating also exhibited antibacterial properties by effectively reducing bacterial adherence and proliferation of *E. coli* and *S. aureus* strains, indicating its dual functionality as a scaffold for bone tissue regeneration and infection prevention.

Although pure zein has limited hydrolytic stability and mechanical strength, these shortcomings are gradually addressed through composite reinforcement, bioactive loading and structural optimisation [29,37,45]. Zein’s compatibility with both hard (e.g., hydroxyapatite) [12,37] and soft (e.g., PCL, gelatin) [29,50] biomaterials facilitate their integration into various scaffold systems, broadening their applications from osteogenic differentiation and regeneration to antimicrobial wound healing [34].

#### 2.1.3. Soy Protein

Soy protein, derived from the legume family of plants, is primarily composed of globulins and albumins. This is noteworthy from the standpoint of BTE due to their rich amino acid profile, which has been associated with improved biocompatibility and biodegradability. Rich in both acidic (aspartic, glutamic) and basic (lysine, arginine) amino acids, soy protein supports a biological environment conducive to cell proliferation and differentiation [33]. Its favourable cytocompatibility and low immunogenicity, combined with the absence of zoonotic transmission risks associated with animal-derived proteins, make soy protein a promising green alternative for biomedical applications [33,51].

The most utilised soy form in scaffold development is soy protein isolate (SPI). SPI shows excellent bioactivity but has poor solubility and weak mechanical strength in its native hydrogel form, which significantly limits its standalone use in BTE [52]. The complex and heterogeneous protein structure of SPI contributes to difficulties in forming chemically crosslinked networks, while physically crosslinked SPI hydrogels often display insufficient water resistance and durability under physiological conditions [35]. To address these limitations, various strategies have been proposed, including polymer blending and the use of reinforcing nanomaterials.

One such approach is the fabrication of composite microcarriers combining SPI with chitosan. A recent study evaluated SPI-impregnated chitosan (CS) microcarriers, specifically 7S, 11S and full SPI variants, for their ability to support the growth and differentiation of rat adipose-derived mesenchymal stem cells (rADSCs). All composite microcarriers presented a porous 3D architecture, with 11S/CS and 7S/CS formulations demonstrating significantly improved porosity and surface potential compared to controls. These carriers enhanced rADSC adhesion, proliferation, and osteogenic differentiation, as evidenced by elevated ALP activity, collagen type I (COLI) expression, and mineralisation (via Alizarin Red S staining), thus confirming SPI’s role in promoting osteogenesis [51].

To further expand the functional utility of SPI, 3D printing has emerged as a powerful strategy to fabricate scaffolds with controlled architecture and tunable mechanical properties. Dorishetty et al. successfully developed a hybrid hydrogel system through photochemical crosslinking of globular SPI with fibrous silk fibroin (SF) [35]. The 3D-printed SPI/SF hydrogels displayed significantly enhanced mechanical strength (Young’s modulus: 214–811 kPa) and pore diameter of 45.52 ± 3.56 μm compared to casted controls (5.97 ± 1.18 μm), alongside robust fibroblast cell attachment and proliferation. While this study focused on fibroblasts, the established printability and tunable stiffness of the SPI/SF hydrogels suggest strong potential for BTE, particularly when paired with osteogenic cell types in future studies.

These findings establish the potential of soy protein, particularly SPI, as a sustainable and biocompatible platform for bone scaffold development. Despite inherent limitations in mechanical strength, advancements in composite formulations (e.g., with chitosan or silk fibroin) and fabrication techniques (e.g., 3D printing, microcarrier systems) have demonstrated its potential in supporting MSC activity, osteoblast function, and mineralised matrix formation, underscoring its viability as a plant-based scaffold material in BTE.

### 2.2. Polysaccharides

Polysaccharides are widely used in tissue engineering for their biocompatibility, biodegradability and low cost, along with their structural resemblance to the ECM. They are broadly classified into structural polysaccharides (like chitin, cellulose) and storage polysaccharides (starch, glycogen). However, challenges such as variability in branching and molecular weight can affect scaffold uniformity and performance [53]. In this Section, we focus specifically on plant-derived polysaccharides, namely, cellulose, starch and gums, and their applications in BTE.

#### 2.2.1. Cellulose

Cellulose is one of the most abundantly found and structurally versatile plant polysaccharides that has emerged as a promising choice for BTE owing to its mechanical strength and biocompatibility. Structurally, it is constituted of repeated β-1,4-linked D-glucose units and is synthesised by plants, bacteria (*Acetobacter xylinum*, *Pseudomonas* spp.), fungi, algae and marine invertebrates [54,55,56]. The high density of surface hydroxyl groups allows extensive H-bonding, resulting in a semi-crystalline structure characterised by significant mechanical strength, thermal stability and modifiability [22,57]. Although hydrophilic in nature, cellulose in its native form is not soluble in water or conventional organic solvents due to numerous strong intra- and inter-molecular hydrogen bonds between individual chains [58].

Cellulose possesses several essential characteristics for regenerative applications from a biomedical standpoint: besides being biocompatible, biodegradable, it is also non-toxic and non-immunogenic. These attributes promote its integration with living tissues while reducing immune rejection [56,59]. It is worth making a note of the slow degradation rate of cellulose in vivo that has been reported to be over 60 weeks in rat models in the past, and thus methods like oxidation have been used to enhance the rate of degradation, likely due to the numerous strong intra- and inter-molecular hydrogen bonds mentioned above [60]. The simplicity of surface functionalisation enhances its cell adhesion and proliferation capacities, further reinforcing its role as an ECM-mimicking material in scaffold design [57].

Recent advancements in nanotechnology have further augmented the function of cellulose in BTE. Nanostructured materials, such as cellulose nanocrystals (CNCs), nano-fibrillated cellulose (NFC), bacterial cellulose (BC), and TEMPO-oxidised cellulose nanofibres (TOCNFs), exhibit high surface area, tunable porosity, and remarkable mechanical properties, making them suitable for complex scaffold designs [17,22,57]. These nanocellulose materials can be fabricated into fibres, membranes, films, and hydrogels, which are particularly advantageous for replicating the hydrated environment of the native tissue [61].

One such hydrogel-based approach was demonstrated by Im et al. [62], who developed 3D-printable nanocomposite bioinks incorporating tempo-oxidised cellulose nanofibrils (TOCNFs) into alginate matrices. The TOCNFs enhanced both rheological attributes and mechanical strength, optimising the bioink for osteoblast printing and differentiation. A 1.5% concentration of TOCNF proved optimal for cell survival and osteogenic activity, highlighting the capacity of nanocellulose additives to improve printability and influence cell fate in engineered constructs.

In another material innovation, Luo et al. engineered a mineralised cellulose scaffold incorporating calcium and zinc ions, utilising oxidised bacterial cellulose to enhance charge-based mineral binding [63]. The resultant ZOBNS scaffold not only mimicked ECM-like structures but also functioned as a reservoir for Ca^2+^ and Zn^2+^, resulting in enhanced osteogenic gene expression (*RUNX2*, *OCN*, *COL-1*) and higher BM MSC proliferation. These findings highlight the ability of cellulose to serve as both a structural and biochemical platform, boosting bone tissue regeneration via ion-mediated signalling.

The fabrication method also plays a crucial role in optimising scaffold performance. Liesiene et al. synthesised rigid cellulose gels via the slow hydrolysis of cellulose acetate in an acetone–aqueous ammonia environment [61]. This approach enabled the re-formation of H-bonding networks, which further resulted in stable, porous 3D structures after lyophilisation. Moreover, the hydrogels exhibited remarkable mechanical properties, with a compressive (Young’s) modulus of ~43 MPa and an elastic modulus of up to 0.23 MPa. These gels supported the vascularisation and bone ingrowth, thereby highlighting the significance of both microarchitecture and material chemistry in scaffold efficacy.

All of these developments collectively demonstrate the evolution of cellulose from a structural polysaccharide to a multifunctional scaffold material [22,57]. The advancement of cellulose-nanocomposite hydrogels, 3D-printable bioinks [62] and ion-loaded mineral scaffolds [63] exemplifies the modularity and tunability of cellulose platforms in tissue engineering. Importantly, the adaptability of cellulose allows it to interface effectively with other bioactive components such as alginate, polydopamine nanoparticles (PDANPs), or metallic ions to enhance both mechanical properties and osteogenic outcomes [57,62,63].

#### 2.2.2. Starch

Starch is a naturally occurring carbohydrate polymer composed primarily of two glucose-based polysaccharides: amylose, a mostly linear polymer with α-(1→4) linkages, and amylopectin, a highly branched polymer featuring both α-(1→4) and α-(1→6) glycosidic bonds. These macromolecules form semi-crystalline granules whose structure, crystallinity and morphology vary based on the plant source [64,65]. The complex structure of starch is one of its key traits in scaffold materials for BTE, contributing to its biocompatibility, biodegradability and hydrophilicity [30,64,65]. Its hydrophilic nature not only supports cellular adhesion and proliferation but also promotes the scaffold degradation and eventual integration with the host tissue [66,67,68]. Nevertheless, native starch exhibits low mechanical strength, brittleness and sensitivity to moisture, which limits its individual application in tissue repair. Consequently, recent strategies have focused on enhancing its properties through composite formulations with other polymers, nanoparticles and ceramics to broaden its functional applicability in BTE [30,67,69].

A study by Mirab et al. demonstrated how structural tuning of starch-based scaffolds can meet physiological requirements for bone regeneration [67]. The team used unidirectional freeze-casting followed by freeze-drying, and a starch/PVA scaffold was engineered with a gradient pore structure ranging from 80 to 292 μm, which is ideal for cell infiltration and vascularisation. The incorporation of cellulose nanofibres and hydroxyapatite (HA) nanoparticles not only improved compressive strength but also enabled mineral deposition through enhanced nucleation. The scaffold showed >94% cytocompatibility with MG-63 osteoblasts, thereby confirming its safety and biological functionality.

In parallel, enhancing the osteogenic potential and mechanical resilience of starch-based scaffolds has also been pursued through carbon nanomaterial integration. Asl et al. fabricated electrospun nanofibres composed of polyhydroxybutyrate (PHB) blended with starch and multiwalled carbon nanotubes (MWCNTs) [69]. The inclusion of starch in the blend led to improved hydrophilicity and biodegradability, creating a more favourable environment for the MG-63 cells. Added to this, the MWCNTs enhanced mechanical strength (tensile strength > 24 MPa) and surface roughness, promoting osteo-conductivity. The composite scaffold containing 1 wt% MWCNTs significantly upregulated key osteogenic markers, such as *COL1*, *OCN*, *OPN*, and *osteonectin (ON)*. This is attributed to the synergistic effect of the cell-supportive starch surface in addition to the bioactive and structural contributions of MWCNT. This improved scaffold morphology also facilitated calcium phosphate deposition, an indication of early bone formation.

Further addressing the mechanical shortcomings of starch, Taherimehr et al. explored the potential of thermoplastic starch (TPS) reinforced with β-TCP [30]. TPS is produced by plasticising native starch with glycerol, enhancing its processability. The TPS/β-TCP composites, processed via extrusion and injection moulding, exhibited uniform β-TCP dispersion and improved bioactivity. This was established by the hydroxyapatite layer formation in simulated body fluid (SBF). These scaffolds also demonstrated high compatibility with human MSCs, with viability exceeding 97%.

To better understand the synergistic effect of blending starch with other biodegradable polymers, Asl et al. explored PHB–starch blends for BTE applications. Their results revealed improved hydrophilicity, mechanical strength and scaffold degradation rates [65]. The PHB-10% starch formulation presented optimal properties: reduced fibre diameter, improved thermal stability and significantly enhanced ALP activity and mineralisation in MG-63 cells. The porous architecture of the scaffolds and surface energy facilitated enhanced cellular attachment and osteogenic differentiation.

These studies position starch-based scaffolds as versatile, sustainable and biologically active platforms for bone regeneration. There are challenges to using starch, including moisture sensitivity and variability in mechanical integrity; however, material modifications via nanofillers, hybrid polymers and mineral reinforcements are promising solutions to these challenges. Their consistent biocompatibility and performance in osteoblast and MSC cultures indicate strong potential for preclinical translation, particularly for non-load-bearing and supplemental BTE applications.

#### 2.2.3. Alginate

Alginate is one of the most widely available biomaterials, mainly from brown seaweed and bacteria. It contains a thousand blocks of β-d-mannuronic acid (M) and α-l-guluronic acid (G) monomers connected via a 1→4 linkage [70]. The different blocks of alginate are arranged consecutively as G residues (GGGGGG), then consecutively as M residues (MMMMMM), and then interchanging G and M residues (GMGMGM). The content of the G-block in the Laminaria hyperborean stems is about 60%, whereas in other commercially obtainable alginates, it has a range of 14.0–31.0% [71].

It is known for its biocompatibility, biodegradability, non-toxicity, flexibility and chelating ability. It is hygroscopic but has low solubility and poor degradability. To address these issues, it is often used in conjunction with other materials to enhance these properties. The fabrication of alginate gels has enabled the enhancement of some of these properties for BTE applications [70]. The weight average molecular weight of the SMWA is 16,190 g/mol, while that of the original alginate is 166,700 g/mol [72].

Mejuto and Gonzales fabricated 3D-printed alginate–hydroxyapatite aerogel scaffolds and evaluated the attachment and proliferation of MSC on these scaffolds for BTE. They found that the resultant scaffolds were highly porous (80%) with pore diameters ranging from 19 ± 1 to 31 ± 2 nm, encouraged cellular attachment, cell viability and proliferation along with fibroblast migration towards damaged areas. The authors concluded that these properties made these alginate scaffolds suitable for BTE applications [73]. Zhou et al. worked with alginate hydrogel as a carrier of the CB2 Agonist JWH133 for Bone Engineering and found that the resultant hydrogel had a high drug-loading capacity, was biocompatible, and had strong potential as a drug carrier for treating osteoporosis by promoting osteoblast and inhibiting osteoclast formation and function [74].

Eskandani et al. fabricated electroconductive nanofibrous oxidised alginate scaffolds using electrospinning with PVA for BTE applications. Their results indicated high hydrophilicity, biocompatibility with the MG-63 cells, and with an average pore size of 2.3–2.5 μm, which were well suited for BTE applications [75]. Taken together, along with the right combination, alginate is a suitable natural polymer that can be used for BTE applications.

#### 2.2.4. Gums

Plant-based gums are water-soluble polysaccharides that are commonly used as thickening agents and stabilisers in food and pharmaceutical applications. They are naturally occurring plant, microbial or algal polysaccharides, gaining prominence in BTE mainly for their biocompatibility, water retention, absorption and ease of chemical modifications [76]. Their ability to form hydrogels makes them particularly attractive for mimicking the hydrated ECM, offering a conducive environment for cell adhesion, proliferation and differentiation.

Mirza et al. developed a ternary nanocomposite scaffold incorporating nano-hydroxyapatite (n-HA), gum arabic (GA) and κ-carrageenan (κ-CG) [77]. The CHG2 formulation (60/20/20 ratio) showed superior apatite layer formation in SBF, enhanced compressive strength (9.2 ± 1.1 MPa), compressive modulus (567 ± 2.5 MPa) and elevated expression of osteogenic markers such as *osteocalcin*, *osteonectin* and *osteopontin.* This underscores the synergistic bioactivity of GA and κ-CG with HAP in promoting bone regeneration.

Similarly, Lett et al. fabricated porous HAP scaffolds using gum ghatti (GG) as a natural binder [78]. These scaffolds exhibited interconnected micro- and macroporosity (200–500 μm), mechanical stability, and encouraged cell compatibility with MDCK cells, suggesting that GG can serve as a green binder to support scaffold architecture and biological integration in orthopaedic applications.

Expanding on hydrogel applications, Kim et al. synthesised phosphate-crosslinked guar gum (GG) hydrogels that showed efficient biomineralisation, enhanced MC3T3-E1 cell proliferation and apatite formation with a Ca/P ratio mimicking natural bone [79]. Interestingly, increasing GG content improved porosity, ranging from 62 to 81%, and cell viability while slightly reducing mechanical strength, indicating a trade-off that must be tuned based on application.

Together, these studies show that natural gums such as gum arabic, gum ghatti, guar gum, tragacanth gum and κ-carrageenan can be effectively integrated into scaffolds or hydrogels to enhance both mechanical and biological performance in BTE, reinforcing their value as sustainable, green biomaterials.

## 3. Animal-Based Natural Polymers for BTE

### 3.1. Proteins

Animal proteins like silk fibroin and gelatin are well known for their industrial and biomedical applications in tissue engineering. While most of these materials are used in combination with stronger materials for BTE to enhance their mechanical strength, each of these proteins provides unique advantages. For example, collagen provides an appropriate environment for bone formation and regeneration as it forms a major part of the bone organic matrix. Silk fibroin offers biocompatibility, biodegradability and overall versatility of fabricating into different forms. Gelatin encourages cell growth, proliferation and is easily combined with other materials for the fabrication of scaffolds of BTE. This Section explores these materials for BTE applications.

#### 3.1.1. Collagen

Collagen is the most abundant structural protein in animals and a primary component of the ECM, where it plays a key role in providing mechanical strength, tissue architecture and biochemical cues. It constitutes nearly 25% of the total protein content in mammals and forms the structural foundation of hard tissues such as bone and dentin [80,81]. In bone, type I collagen accounts for ~90% of the organic matrix and serves as the primary scaffold for mineral deposition, particularly of hydroxyapatite (HA), thus directly influencing bone regeneration and remodelling [82].

Collagen scaffolds have been widely utilised in BTE due to their biocompatibility, biodegradability and low immunogenicity. Their porous, fibrillar architecture mimics native ECM and facilitates cell adhesion, migration and differentiation, while also supporting vascularisation and nutrient exchange, all essential features for successful tissue integration [53]. Moreover, collagen exhibits both osteoconductive and osteogenic properties and has been explored extensively as a carrier for bioactive molecules, such as bone morphogenetic proteins, to further enhance osteogenesis [83].

Despite these advantages, collagen scaffolds suffer from limited mechanical strength, especially in load-bearing scenarios. To address this, crosslinking (chemical, enzymatic or physical) and composite reinforcement with ceramic or synthetic phases are often employed to enhance structural integrity and prolong scaffold degradation time [84].

One notable approach has been the development of biomimetic mineralised collagen (BMC) scaffolds, designed to replicate the composite structure of natural bone, consisting of collagen fibrils interspersed with inorganic mineral crystals. As highlighted by Wu et al., BMC scaffolds have shown promise due to their favourable mechanical properties, high bioactivity and compatibility with MSCs [85]. These scaffolds closely resemble native bone ECM both in composition and microstructure, making them highly effective in supporting osteoblast differentiation and new bone matrix deposition.

Another innovation in scaffold fabrication involves 3D printing of collagen-based materials, which allows for high-precision customisation of pore size, architecture and cell distribution. Guo et al. developed a 3D-printed collagen/HAP scaffold using a gelatin support bath to address collagen’s poor printability [86]. This hybrid system, which incorporated BM MSCs, demonstrated high cell viability, elevated ALP expression and stable mechanical performance. The collagen/HAP composite exhibited suitable rheological properties for extrusion printing, had a pore size of 500 × 800 μm and was able to retain its 3D shape, promoting osteogenic differentiation. However, the authors also noted limitations in replicating microstructural complexity, emphasising the need for further development in high-resolution 3D bioprinting for microarchitecture guidance.

Complementing this, Santhakumar et al. explored the surface mineralisation of collagen scaffolds by comparing two types of coatings: amorphous calcium phosphate (ACP) and low-crystalline apatite [87]. Their results, obtained from a rat cranial defect model, revealed that only the apatite-coated collagen (Col-Ap) improved in vivo bone regeneration significantly, whereas the ACP-coated scaffold (Col-ACP), despite showing bioactivity in simulated body fluid, failed to support effective bone healing. This highlights a critical insight: the stability and crystallinity of the mineral coating play a crucial role in determining scaffold performance in vivo, and not all in vitro mineralisation processes reliably translate to regenerative efficacy.

Building on the bioactivity of mineralised collagen, Gharati et al. introduced a collagen hydrogel nanocomposite enriched with 2% strontium (Co/BGSr2%) and seeded with MSCs in a rabbit bone defect model [88]. This combination significantly enhanced bone regeneration, as evidenced by histological scores, radiographic density, and osteocalcin expression. The interplay between collagen’s biocompatible matrix and the osteoinductive influence of strontium ions effectively enhanced the scaffold’s capacity to stimulate rapid bone regeneration. These findings underscore the potential of trace-element-enriched collagen composites to fine-tune cellular responses during regeneration.

While most of the studies discussed focus on BTE, the potential of collagen in cartilage regeneration also demonstrates its broad applicability in musculoskeletal TE. Intini et al. developed a composite scaffold combining Type I and II collagen with hyaluronic acid (HyA), with the size of collagen fibres close to 100 nm [89]. They observed an enhanced sulphated glycosaminoglycan (sGAG) deposition and also the promotion of MSC chondrogenic differentiation. These results affirm the versatility of collagen and reinforce the value of mimicking the biochemical composition of target tissues to optimise regenerative outcomes.

The reader is also directed to a comprehensive review by Li et al. that provides a detailed overview of mineralised collagen scaffolds [90]. The study detailed fabrication methods, like direct mineral incorporation, in situ mineralisation, and 3D printing and their dependence on biophysical and biochemical cues. The review emphasised how scaffold mechanics, surface chemistry, and topography regulate MSC behaviour, further validating the biomimetic strategy of combining collagen with minerals for improved osteogenesis.

In summary, the natural origin, structural compatibility with bone ECM, and tunable properties via mineralisation and crosslinking make collagen a versatile and potent material for bone tissue scaffolds. Innovations in 3D printing, nanocomposites and ion supplementation are actively addressing its mechanical and functional limitations.

#### 3.1.2. Silk

Silk fibroin (SF), predominantly sourced from the silkworm *Bombyx mori*, has attracted considerable interest in BTE due to its biocompatibility, mechanical strength, processability and tunable degradation [91,92]. The structural complexity is attributed to its composition: a heavy chain (~390 kDa), a light chain (~26 kDa) and the glycoprotein P25, which collectively establish a hierarchical structure reinforced by β-sheet crystallites, van der Waals forces, and hydrophobic interactions [91,93]. These characteristics provide SF with significant tensile strength and viscoelasticity, facilitating its use in various scaffold formats such as hydrogels, sponges, electrospun fibres and 3D-printed structures, each suited to specific bone defect geometries [93,94,95].

Despite all these advantages, SF alone frequently lacks the necessary osteo-inductivity and mechanical strength for orthopaedic applications. Bosio et al. emphasised that pure SF scaffolds generally demonstrate a significantly lower Young’s modulus than native bone and are deficient in adequate osteogenic signalling [96]. To address these limitations, the authors created hybrid SF scaffolds that integrate nano-structured CaCO_3_ microparticles. These composites improved mechanical strength, mineralisation and osteogenic gene expression, while also facilitating co-cultures of hMSCs and THP-1-derived osteoclasts. The incorporation of CaCO_3_ particles facilitates dual functionality in drug delivery.

Addressing the need for structural reinforcement, Braxton et al. created a biphasic scaffold that integrated 3D-printed PEGT/PBT with SF infilling with a wide distribution of pore sizes ranging from 15 to 370 μm [95]. The rigid lattice maintained mechanical stability, while the SF layers offered biocompatibility, surface area and porosity suitable for osteochondral tissue regeneration. This integration facilitated customised scaffold characteristics in both cartilage and bone areas, underscoring the promise of personalised scaffold design through additive manufacturing.

In another approach, Spessot et al. developed methacrylated SF scaffolds by a method that integrates salt leaching and UV-initiated crosslinking [97]. The dual-mode fabrication enhanced mechanical strength and structural uniformity, thereby reducing significant limitations of native SF and exhibiting compatibility with bone tissue settings.

Norouzi et al. investigated the formulation of a 3D bio-printable SF–alginate–gelatin bioink, augmented by the incorporation of alendronate [94]. The incorporation of SF enhanced hydrogel durability and printing accuracy, while facilitating osteogenic differentiation by elevating alkaline phosphatase activity in MG-63 cells, with a pore size ranging from 472 to 837 μm. This study demonstrated SF’s role not only as a structural element but also as an active participant in regulated medicinal delivery.

Expanding beyond *Bombyx mori*, Lee et al. explored the combination of spider SF and carboxymethyl cellulose (CMC), extending their research beyond *Bombyx mori* [98]. The exceptional tensile strength and fatigue endurance of spider silk, along with the enhanced porosity from CMC, resulted in scaffolds that facilitated cell infiltration and nutrient exchange, demonstrating increased osteogenic potential. This suggests that non-mulberry silk sources may provide distinct mechanical and biological characteristics for particular BTE applications.

Collectively, these research studies illustrate the innovations being undertaken to overcome the constraints of native SF by chemical functionalisation, composite formulation and optimisation of scaffold architecture. SF is increasingly recognised as a versatile platform in BTE, with applications ranging from 3D-printed hybrid systems to mineral-loaded sponges and responsive bioinks.

#### 3.1.3. Gelatin

Gelatin is a natural biopolymer derived from the partial hydrolysis of collagen and is a widely used material in BTE for its structural similarity to the ECM, biocompatibility, and abundance of functional groups that facilitate chemical modification [99]. It has the crucial arginine–glycine–aspartate (RGD) sequence, which is critical for integrin-mediated cell adhesion [100]. It is an essential factor in the attachment, proliferation and osteogenic differentiation of bone-forming cells such as osteoblasts and MSCs [100]. However, in spite of these biological features, gelatin has poor mechanical properties and rapid biodegradation under physiological conditions. This limits its use as a standalone scaffold material in bone repair applications [81].

To overcome these challenges, recent studies have focused on composite scaffolds and advanced fabrication techniques. For instance, Gautam et al. fabricated electrospun gelatin–PCL scaffolds incorporated with nanohydroxyapatite (nHA) [99]. This nanocomposite scaffold with an average fibre diameter of 615 ± 269 nm and average pore size of 4.7 ± 1.04 μm, supported high osteoblast viability and proliferation, with cells exhibiting well-spread polygonal morphology, which is an indicator of active osteoblast phenotype. These findings were supported by MTT and DNA quantification assays, suggesting that such composite scaffolds effectively promote cellular activity necessary for bone regeneration.

Another promising strategy involves blending gelatin with substances like CS, a natural polysaccharide structurally similar to glycosaminoglycans found in native bone ECM. Bozorgi et al. developed chitosan–gelatin (CS/Gel) scaffolds that supported not only pre-osteoblast attachment and viability but also significantly enhanced the osteogenic differentiation of MSCs [101]. The inclusion of copper-substituted nanohydroxyapatite (Cu-nHA) improved scaffold strength and mineralisation and reduced porosity from 99.555 ± 0.394% to 98.69 ± 0.80%.

To further improve the functional versatility of gelatin, Bhushan et al. incorporated cerium oxide nanoparticles (CNPs) into chitosan–gelatin scaffolds [81]. These CG-CNP nanocomposites exhibited suitable compressive strength and apatite-forming capacity, both critical for bone repair. Notably, they also demonstrated antimicrobial properties and cytocompatibility in vitro, as well as osteo-conductivity in vivo CAM assays. These features position gelatin-based composite scaffolds as strong candidates for clinical use, where infection control and early-stage mineralisation are essential.

Beyond material composition, fabrication methods critically influence scaffold architecture and function. Electrospinning remains a key technique for producing nanofibrous scaffolds that mimic the native ECM, as demonstrated in the gelatin–PCL–nHAp study. Meanwhile, 3D printing offers precise control over scaffold geometry and porosity, essential for matching irregular bone defects. Kara et al. highlighted gelatin’s printability, tunable rheological properties and the benefits of enzymatic crosslinking (e.g., microbial transglutaminase), which yielded scaffolds with enhanced porosity, mechanical strength and osteo-conductivity [102].

Gelatin serves as a highly adaptable polymer in BTE. Its biological affinity, when combined with other synthetic polymers, bioceramics and smart nanoparticles, enables the design of scaffolds that support critical osteogenic processes. These advances, alongside scalable and precise fabrication techniques, continue to position gelatin-based scaffolds at the forefront of bone regenerative strategies.

### 3.2. Polysaccharides

#### 3.2.1. Chitosan (CS)

Chitin is a natural polymer mainly derived from marine sources from the shells of crustaceans, as well as from the walls of fungi [22,103]. Once extracted, chitin is then subjected to deacetylation of at least 60% to obtain CS. It is polyelectrolyte and semi-crystalline in nature, and homopolymeric chitosan consists of randomly placed N-acetyl-D-glucosamine and D-glucosamine repeating units linked by β-(1-4) glycoside bonds [104,105,106]. The process of deacetylation involves the removal of acetyl groups from the molecular chain of chitin, leaving behind a complete amino group (–NH2) to form chitosan [107,108]. Evidence suggests that a higher degree of deacetylation and lower molecular weight of CS has demonstrated higher antimicrobial, anti-inflammatory, anti-oxidant, as well as anti-cancer properties [109].

To enhance its mechanical properties for BTE, chitosan is often formulated as a scaffold or a membrane alongside other materials like polyvinyl alcohol (PVA), bio-ceramics and PCL using various methods like electrospinning, chemical precipitation and solvothermal techniques [107,110]. Vaidyanathan and colleagues fabricated CS/silver composite scaffolds for BTE applications with pores greater than 100 μm in diameter and found the scaffolds to be highly biocompatible, supportive of osteoblast growth and exert broad-spectrum antibacterial activity [111]. Yousefiasl et.al worked with CS/alginate bio-nanocomposites with mesoporous silica nanoparticles for BTE applications with pore sizes ranging from 119 to 221 μm. Their study found that this combination of scaffolds not only enhanced rat BM MSC viability but also significantly improved the biomineralisation properties, compared to the control [112].

While the majority of the CS is still acquired from marine sources, a recent study compared CS from both marine and fungal sources for differences in physicochemical and biological properties. Their formulations were in combination with β-TCP, ranging from 0, 10, 20, to 30% with animal or fungal CS [103]. Their results indicated that fungal-derived CS had similar properties to the widely used marine sources and can be used as an alternative to the animal-derived chitosan for BTE applications. Yildizbakan and colleagues fabricated a CS/cerium oxide porous scaffold (pore size ranging from 0 to 160 μm) with antibacterial properties and found that their scaffold manifested negligible cytotoxic effects and exhibited inhibitory effects on bacterial growth against *Staphylococcus* aureus and *Escherichia coli* strains [16]. Collectively, the literature presents encouraging evidence supporting the use of chitosan in BTE, owing to its diverse beneficial properties.

#### 3.2.2. Hyaluronic Acid (HA)

Hyaluronic acid (HA) is a naturally occurring polysaccharide found throughout the human body and forms a major component of the musculoskeletal system, in the cartilage, synovial tissue, among others [113]. It is a non-sulphated glycosaminoglycan (GAG) that has witnessed an increased interest in hydrogel formation for BTE applications [114]. This has been due to their swelling properties, along with their biodegradability, biocompatibility and for their unique ability of cellular movement and proliferation via the CD44 surface receptor signalling [114]. Due to the fact that HA naturally exists inside the human body and the musculoskeletal system, it makes HA a promising biomaterial for BTE. However, for BTE, it is often utilised with other materials to enhance its mechanical strength and durability [114,115].

Asensio and colleagues fabricated a biomimetic scaffold with interconnected macropores (>100 μm) and micropores (<10 μm) for osteochondral tissue engineering using HA with strontium and zirconium folates and performed physico-chemical testing, in vitro and in vivo evaluation. Their study found that their scaffold had high swelling capacity and low degradation rate, low toxicity rate towards human osteoblasts and cartilage cells. The scaffolds also promoted guided cell proliferation in vitro and tissue regeneration in vivo [116]. Liu et.al prepared scaffolds for BTE on aligned poly (lactic-co-glycolide) (PLGA) nanofibres ranging from 108 to 299 nm in fibre diameter, incorporated with hyaluronic acid oligosaccharide–collagen mineralised microparticles (labelled oHA-Col/HAP). Their scaffolds provided an appropriate environment for encouraging the migration and recruitment of osseointegration-related cells and were stimulatory of cell proliferation. [117].

Yun et.al investigated the bone volume (BV), tissue volume (TV) and BV/TV ratio in vivo in New Zealand white rabbits using a control group, a group with 3D-printed polylactic acid (PLA) scaffolds with or without hyaluronic acid (HA), i.e., 3D PLA and 3D PLA/HA. While there was no statistical difference in bone formation across the three groups, the highest BV/TV ratio was observed in the scaffold group with HA (3D-PLA/HA) group at 12 weeks. Based on the findings, researchers concluded that 3D-printed PLA/HA scaffolds have the potential to enhance bone augmentation [118]. Interestingly, a meta-analysis of three studies on the use of HA in BTE in 2024 suggested that while these three studies indicate promise towards bone formation by the addition of HA to bone grafts, these changes were not statistically significant. However, it must be noted that their final analysis had only three investigations [119].

## 4. Innovation and Sustainability in Biomaterials for BTE Applications

**Figure 3:**
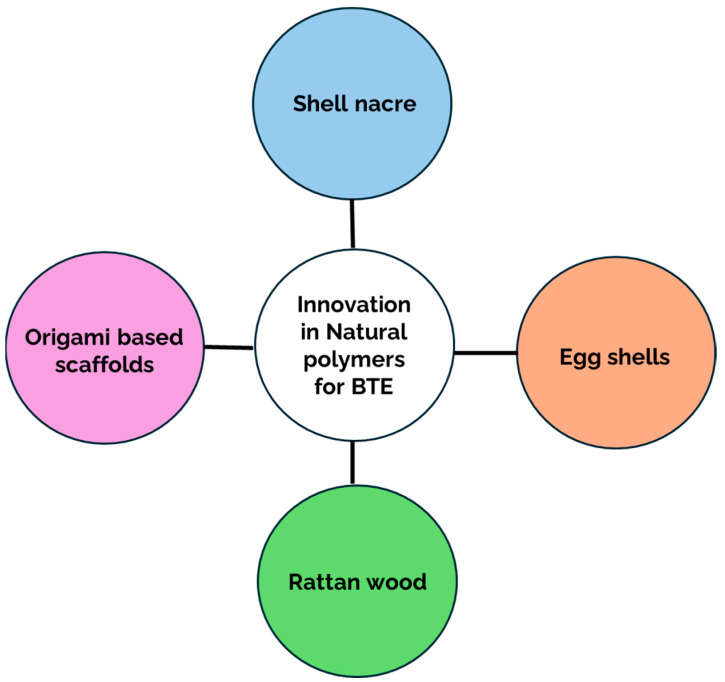
Examples of innovative materials and sustainable natural polymers for bone tissue engineering (BTE) applications.

Apart from the commonly known and used natural polymers for BTE, the last couple of years have witnessed an increase in novel and innovative materials that also provide a sustainable approach towards BTE applications. Some of these include rattan wood, egg shells, marine sources and origami-based approaches (Figure 3), and are outlined in Table 1.

### 4.1. Rattan Wood

BTE increasingly aims for biomaterials that not only promote osteogenesis and integrate effectively with host tissue but also replicate the mechanical and structural intricacies of native bone. In this regard, rattan wood (*Calamus manan*) has emerged as a particularly interesting natural resource to produce biomorphic, 3D scaffolds [27].

The appealing feature of rattan is its highly anisotropic and porous microarchitecture, consisting of aligned fibre bundles and longitudinal channels, which is structurally comparable to the osteon system of compact bone [120]. This hierarchical structure originates from the wood’s inherent composite composition—cellulose, hemicellulose and lignin—which together provide rigidity, elasticity and durability [121]. This intrinsic architecture, when used as a biomorphic template, can be chemically transformed into bioceramic scaffolds through a pyrolysis-initiated gas–solid reaction sequence that maintains the wood’s microstructural complexity while converting it into nanocrystalline, ion-doped hydroxyapatite (B-HA) [27].

Through a distinctive physical and chemical process, the rattan wood is transformed biomorphically into a bone substitute that aids in the regeneration of host bone [120,121,122]. This innovative biomaterial has robust biocompatibility and bioactivity and has been extensively tested for bone grafting and regenerative therapies [25,123]. The human bone is a porous bio-hybrid composite, primarily composed of hydroxyapatite (70 wt%) and collagen (30 wt%). Hydroxyapatite bone scaffolds, distinguished by their highly organised hierarchical structures, are synthesised through the chemical transformation of native woods via a series of thermal and hydrothermal processes [120].

A leading application of this strategy is the GreenBone scaffold, a CE-marked device developed through a six-step biomorphic transformation of rattan wood. The scaffold retains longitudinal vascular channels (~300 µm) and significant interconnectivity (~60% porosity), facilitating cell migration, nutrient transport and vascular ingrowth, which are necessary for bone regeneration [124,125]. Both in vitro and in vivo studies consistently demonstrated the bioactivity and osteo-inductivity of B-HA, showing its capacity to regenerate bone tissue independently of exogenous growth factors, cells or biomolecules [23,124]. The absence of growth factors in regeneration potential substantially diminishes translational obstacles by simplifying regulatory requirements and storage issues, hence expediting clinical application and lowering healthcare expenses.

Volker Alt et al. summarised the clinical and radiographical outcomes of defect filling of the iliac crest in nine patients using rattan-wood-based not-sintered hydroxyapatite and β-TCP material [126] (commercially known as b.Bone™ and manufactured by GreenBone ORTHO S.p.A, Faenza, Italy) [127]. All nine patients demonstrated successful wound healing at the iliac crest; seven patients reported the absence of pain, while two patients experienced only mild discomfort following an average follow-up period of 9.8 months. There were no postoperative hematomas, surgical revisions or other complications related to the implants at the iliac crest. In all patients, favourable radiographic integration, with no implant dislocation, and satisfactory bone integration were noted, thereby confirming the biocompatibility and biomimetic properties of the implants [126].

In vivo implantation in rabbits validated the ectopic bone formation capabilities of B-HA, whereas ion-doping with Mg^2+^ and Sr^2+^ demonstrated potential in augmenting osteogenic differentiation and antibacterial properties [124]. These ions not only replicate the natural bone microenvironment but also facilitate the rebalancing of bone turnover, rendering B-HA an optimal scaffold choice even in osteoporotic circumstances. The B-HA scaffold’s efficacy in cell-based regenerative therapies has been established. A study reported high attachment rates (~98%) for both cultivated and uncultured human MSCs, with substantial vitality maintained over a four-week period [128]. Notably, the distinct transcriptional signatures observed between cell types, particularly in key osteogenic markers (*RUNX2*, *ALP*), highlight the scaffold’s ability to support cell-specific gene expression dynamics. Furthermore, co-culture with endothelial cells resulted in elevated VEGF secretion, confirming the scaffold’s capacity to promote angiogenesis, a vital component for successful bone integration and vascularised tissue repair.

From a mechanical performance perspective, B-HA scaffolds exhibit unusual toughness for a ceramic and possess a tensile-to-compressive strength ratio greater than one, placing them in a rare zone on the Ashby plot typically unoccupied by ceramics [23]. This damage-tolerant feature is particularly useful in addressing substantial, load-bearing bone deformities, as conventional ceramics frequently succumb to brittleness.

Another case study assessed the clinical outcomes of a bone grafting procedure performed using b.Bone™ combined with bone marrow aspirate concentrates [129]. The patient with the graft in the left distal femoral bone showed favourable post-operative outcomes, with successful bone healing, restorative bone function marked by full weight-bearing capacity, and complete recovery in one year. Therefore, this method serves as a practical substitute for conventional bone grafting techniques, providing structural support, promoting biological healing and eliminating the complications related to harvesting autologous bone grafts [129]. Another study by Salamanna et al. evaluated the efficacy of b.Bone™ in reconstructive surgery in the presence of infection using an in vitro 3D bone fracture model. They supplemented the scaffold with gentamicin or vancomycin and showed that the scaffold exhibited efficient release of potent antibiotics, further validating the potential use of rattan wood in bone surgery and infection [130].

These findings establish rattan-wood-derived scaffolds as a transformative advancement in BTE, integrating biomimetic design, functional ion incorporation and clinical applicability. As the field moves toward more biologically and structurally accurate scaffold models, B-HA offers a compelling solution that bridges the gap between natural architecture and engineering.

**Table 1:** Examples of innovative biomaterials for a green approach to BTE.

No.	Source	Material Component/ Combinations	Scaffold Preparation and Functionalisation	In Vitro/In Vivo Evaluation	Ref.
1.	*Calamus manna* (Rattan wood)	Biomorphic hydroxyapatite (B-HA)	Biomorphic transformation into CaCO_3_, followed by hydrothermal conversion; Doping with Mg^2+^ and Sr^2+^ ions.	Cultured (cMSCs) and uncultured (BMSCs);	[128]
2.	*Calamus* spp. (Rattan wood)	Not-sintered HA, β-TCP	b.Bone™ from biomorphic conversion	Tricortical bone graft harvesting for 9 patients with iliac crest defects	[126]
3.	Origami approach	Wattman filter paper (grade 114)	Wax printing to generate patterns on scaffolds to induce template-guided mineralisation	In vitro MLO-A5 osteoblasts	[131]
4.	*Pinctada maxima* (Silverlip pearl oyster)	Shell nacre orthophosphate composite	Chemical transformation followed by precipitation of carbonate apatite	In vitro MC3T3-E1 osteoblasts and in vivo New Zealand white rabbits	[132]
5.	*Pinctada fucata* (Akoya pearl oyster)	Shell nacre cement (SNC)	Chemical transformation using siloxane methacrylate resin	In vitro evaluations using human BM MSCs	[133]
6.	White leghorn hen eggs	Eggshell membranes sterilised with ethylene oxide	Extraction after acetic acid treatment	In vivo evaluations in adult male Sprague Dawley white rats and adult male white New Zealand rabbits	[134]
7.	Egg shells	Eggshell and egg membrane nanoparticle	Extraction after acetic acid treatment and chemical transformation	In vivo studies in Sprague Dawley rats	[135]

### 4.2. Origami-Based Platforms

Origami-based scaffolds utilise the traditional concept of paper folding, which involves folding sheets into intricate 3D objects. This phenomenon occurs in nature in the form of intracellular protein folding and in the evolutionary structures developed to support winged creatures, leaves and organelles [136]. The origami-inspired implementation provides versatility and flexibility in engineering applications. It can provide voluminous structures that can be compact at the same time and shape-shift to form adaptable 2D or 3D structures [137].

Camci-Unal et al. innovatively developed origami-inspired paper templates that effectively guide osteoblasts’ deposition of calcium phosphate, facilitating template-guided mineralisation [131]. They used Whatman filter paper as the scaffolding material due to its composition of naturally derived cellulose fibres, which is not only biocompatible and flexible but also economical. This material supports cell viability and growth within 3D origami-folded structures. The paper scaffolds were seeded with osteoblasts in collagen and cultured for up to 21 days. To verify the formation of hydroxyapatite minerals, calcium and phosphate staining were performed in conjunction with high-resolution SEM microscopy and elemental analysis. Furthermore, micro-computed tomography (micro-CT) scans played a critical role in determining the distribution of mineralised regions within the origami-folded scaffolds. Therefore, this study serves as a proof of concept for utilising filter paper to fabricate origami-inspired tissue scaffolds aimed at biomineralisation.

Song et al. combined electrospinning technology with origami technique to establish a 3D nanofibre scaffold complex with potential application in BTE. They demonstrated how multilayer nanofibre films served as a scaffold for human foetal osteoblasts (hFOBs) to seed. The secreted extracellular matrix on both sides of the scaffolds facilitated the bonding of adjacent nanofibre films, thereby filling gaps and creating a complete 3D nanofibre scaffold [138]. In an example for another biomedical application, Mei and colleagues created a heart pouch system featuring a distinctive origami design using a memory-shaped microfabricated lattice structure (0.4 μm pore size) that permits effective, repeated and localised cell delivery via minimally invasive surgery [139].

This origami-structure-inspired sealed pouch was tested on a rodent model of myocardial infarction with promising results, as evidenced by the maintenance of viable mesenchymal stem cells along with the sustained release of growth factors and exosomes. The functionality of this model was analysed in vitro as well by Altunbek and Camci-Unal, who cultured the pouch and assessed the release of growth factors and the viability of MSCs at different time points. They were able to confirm that this model was able to secrete hepatocyte growth factor (HGF), fibroblast growth factor (FGF), insulin-like growth factor (IGF) and vascular endothelial growth factor (VEGF) in the cultured medium till the day after which MSCs had to be replenished [140].

The method has been reported to be amongst the most economical approaches for scaffold fabrication, as well as a method that can be used in combination with other methods. Studies directly comparing origami with other methods like 3D printing and electrospinning are currently limited, but results using origami and/or a combination of methods have demonstrated encouraging results. For example, Hossenian et al. combined this with electrospinning, which helped them achieve fine alignment of fibres and control over fibre parameters [141]. Langford and team used 4-D printing in PLA with origami to achieve a significantly higher recovery rate of 96% after compression, in comparison to 61% without origami [142]. Taken together, the concept of using origami to design implants and grafts can be applied to a plethora of biomaterials and offers a high level of adaptability and flexibility in terms of integration into in vivo models.

### 4.3. Materials from Marine Sources

Marine environments are known for their rich biodiversity, and thus biomaterials available from marine sources are also diverse. Sources like seaweed, shells, fish and jellyfishes provide various biomaterials that can be used for tissue engineering [143]. Marine bioresources, including seaweeds, microalgae, mangrove plants, invertebrates, bacteria, fungi and sponges, have been harnessed to create metal and metal oxide nanoparticles [144,145,146]. Specific marine sponges, like *Acanthella elongate*, *Callyspongia diffusa*, *Haliclona* species, *Haliclona exigua* and *Amphimedon* species, have been utilised to synthesise silver nanoparticles and silver nanocolloids. They are noted for producing a wide variety of structurally unique secondary metabolites, such as polyketides, peptides and alkaloids, which demonstrate anticancer, antimicrobial, antifungal and anti-infective properties [147,148,149].

Specifically for bone regeneration, harder materials were first derived from the shells, including the shell nacre of oysters or *Pinctada maxima*. These materials have played an important role towards BTE applications. Ruan et al. investigated nacre-based calcium orthophosphate composite (NCOC) for bone regeneration in vitro and in vivo [132]. In vitro, they found that NCOC demonstrated efficient cell attachment and osteogenic differentiation. Their in vivo investigation using rabbit back fascia indicated enhanced bone healing in a critical bone defect model. Another study investigated shell nacre cement (SNC) for bone regeneration and found that the material would be a suitable candidate for applications in bone void fillings [133].

Ramanjooloo et al. used the marine sponge *Jaspis diastra* to obtain extracts of hexane (JDH), ethyl acetate (JDE) and sterols (JC-2) and formulated a protocol to successfully synthesise AuNPs with the supplementation of contignasterol and ansellone. The exact mechanisms of action of these NPs remain to be elucidated; however, the characterisation and cytotoxicity assays showed promising applications in other biomedical areas like anti-cancer drug development [150]. In BTE, commercially available Coralline algae-derived grafts such as Algipore^®^ and ProOsteon500R^®^ have been extensively used. Algipore^®^ is extracted from *Corallina officialis* and has been documented for its use in bone augmentation, reconstruction and grafting [151,152].

ProOsteon500R^®^ is also a bio-hydroxyapatite-based graft that has been applied in bone enhancement or grafting procedures [153]. In recent years, many advances have been made to extract and characterise marine-based biomaterials in the context of BTE-based applications. Vincent et al. characterised extracts from *Carolline Halimeda* to generate bio-hydroxyapatite. The availability of naturally available hydroxyapatite compound makes this biomaterial non-stoichiometric with a comparable chemical composition to human bone, as it contains trace elements such as Mg^2+^, Si^2+^, Na^+^, K^+^, Zn^2+^, Ba^2+^, F^−^, and CO_3_^2−^. This scaffold can integrate into the bone without toxicity, foreign response or inflammation. In vitro, it also demonstrated enhanced mineralisation, proving its capacity as a reliable and safe biomaterial [154]. Seaweed polysaccharides have also been studied recently as potential scaffolds and hydrogels, as they demonstrate remarkable bioactive diversity and significant therapeutic potential [155]. These properties encompass antioxidant, antitumor, anti-inflammatory, antilipidemic, anticoagulant, antiviral, antibacterial and immunomodulatory functions (reviewed in Jin et al., 2023) [155,156].

### 4.4. Eggshells

Avian eggshell membranes are composed of porous, mineralised tissue, making them an appealing bioresource for scaffold generation. The primary attractive features of this biomaterial include low cost, high availability, biocompatibility, non-cytotoxicity and proven differentiation of stem cells in vitro [134,157,158,159]. Eggshells are composed of a flexible wet-state membrane and a shell which harbours the mineral component. The membrane texture is collagenous and consists of hyaluronic acid, dermatan sulfate, glycosaminoglycans, monosaccharides, lipids and other proteins [160]. Eggshells possess abundant amounts of calcium carbonate (CaCO_3_) along with trace amounts of magnesium carbonate, sodium carbonate, zinc carbonate and phosphate, making them ideal for biomaterial applications such as implants, grafts and wound-healing dressing [161]. 

Yuan et al. tested the biocompatibility of fabricated eggshell membranes with inorganic nanoparticles and showed how this composite scaffold exhibits a fibrous network-like structure, providing a large surface area, good mechanical strength, biocompatibility, and biodegradability in vitro and in vivo in an implantation rat model [135]. Hydrogel-based biomimetic scaffolds have also shown promising results, as demonstrated by Wu et al. Fabricated eggshell microparticle-reinforced gelatin-based hydrogels were assembled to produce mechanically stable and biologically active 3D constructs that can differentiate pre-mature cells into osteoblasts in vitro. These gels were implanted in vivo to assess their biocompatibility, toxicity and durability [162]. Three-dimensional printing has also been explored as an attractive manufacturing technique to formulate bio-composite scaffolds. Gezek et al. supplemented eggshell microparticles to poly(*ε*-caprolactone) (PCL) using 3D printing to achieve bio-compatible, bio-conductive and viable porous scaffolds [163]. In another interesting application, eggshell biowaste was used to generate hydroxyapatite and combined with fibroin extracted from the *Bombyx mori* cocoon.

An injectable hydrogel synthesised using this hydroxyapatite-incorporated fibroin algin was characterised and tested in MC3T3-E1 cultures for its potential application as a dental scaffolding material. The results showed that its high thermostability, low cytotoxicity and favourable half-time of 7 days made this eggshell-based hydrogel a promising biomaterial pending in vivo validation [164]. In conclusion, the biological, physical, and mechanical characteristics of the eggshell membrane render this natural polymer an appropriate fundamental element for the development of novel bone graft materials. Further research is warranted to explore its integration with other pharmaceutical elements for enhanced bioavailability, half-life and biostability.

## 5. Limitations, Conclusions, and Future Directions

The plant- and animal-based biomaterials covered in Section 2 and Section 3 above offer a range of advantages, including low cytotoxicity, flexibility to be formulated into various scaffold forms and biodegradability. However, by themselves, these biomaterials often lack the mechanical strength required for BTE applications and may lack a stable supply across seasons or geographical conditions. Thus, the source materials are often harvested in bulk to extract the biomaterials and used alongside other materials to enhance mechanical strength. While purification of biomaterials, special care must be taken to minimise contamination from antibiotics and microbial waste. Attaining desirable properties for BTE like vascularity and integration within the host tissue can be challenging [20,21]. The novel materials discussed in Section 4 above also exhibit several advantages, but we are yet to know the long-term effect of these materials in vivo. Additionally, to achieve uniformity in scaffold production, several synthesis criteria—like pH, temperature, time, concentration, substrate and medium need to be optimised. This becomes crucial due to the variable parameters of raw materials that interfere with the standardisation of biomaterials, and the possibility of contamination with antibiotics or microbial waste products. As a result, the integration of multiple disciplines will further enhance the field of BTE using natural polymers.

Despite these disadvantages, biomaterials from natural polymers have a promising future in BTE applications. The shift towards a more sustainable approach for material acquisition, processing and scaffold fabrication has witnessed an increase in the use of natural polymers and provides a pathway for a green medicine approach in BTE. They provide an environment that mimics ECM more closely, have superior biocompatibility and are more tunable as per requirements than synthetic polymers [165]. Natural polymers discussed in this review can often be more cost-effective and economic than the production of synthetic polymers and require simplified processes for manufacturing [166]. All of these properties make natural polymers, especially the innovative biomaterials discussed in Section 4 of this review—a promising source for BTE applications.

Looking ahead, it is anticipated that green synthesis methods aimed at a sustainable approach will be utilised widely for BTE applications. Several composite materials are being used with natural polymers to enhance their mechanical properties, and this is predicted to become more common in the near future of biomaterials [167,168,169]. Future and long-term research on eggshell, origami, marine and wood-based materials is required to refine and optimise the technologies to develop consistent (physical and chemical properties), sterile, and long-lasting porous scaffolds. Specifically, how these novel materials compare to the commercial gold standards would enhance our knowledge base to build towards better bone regeneration applications. Standardised sourcing of materials from a single species with controlled diet/nutrition monitoring and housing will help enhance their application in BTE. In addition, combining the innovative biomaterials with different elements to study integration and functionality will further enhance their overall application and performance. In vitro, ex vivo and in vivo evaluations of 3D scaffolds alongside methods like next-generation sequencing will further strengthen our understanding of the effect of natural polymers in physiological conditions. This can provide a pathway to a step in the direction of using 3D platforms for precision medicine [170,171,172].

## Data Availability

No new data were created or analyzed in this study. Data sharing is not applicable to this article.
